# An Improved Bulk DNA Extraction Method for Detection of *Helicoverpa armigera* (Lepidoptera: Noctuidae) Using Real-Time PCR

**DOI:** 10.3390/insects15080585

**Published:** 2024-08-01

**Authors:** Kayla A. Mollet, Luke R. Tembrock, Frida A. Zink, Alicia E. Timm, Todd M. Gilligan

**Affiliations:** Department of Agricultural Biology, Colorado State University, Fort Collins, CO 80523-1177, USA; kayla.mollet@colostate.edu (K.A.M.);

**Keywords:** agriculture, molecular diagnostics, caffeine, invasive species, food security, old world bollworm, *Helicoverpa zea*, bulk DNA extraction

## Abstract

**Simple Summary:**

Old World bollworm is a moth species that causes significant damage to a wide range of agricultural crops. This species was once confined to the eastern hemisphere but has recently spread throughout South America and the Caribbean and has the potential to establish in North America. Molecular detection methods for Old World bollworm have been developed to track its spread and rapidly differentiate it from the native sibling species, corn earworm. Currently, droplet digital PCR (ddPCR) is a preferred method for bulk screening as it is highly accurate and tolerant of PCR inhibitors; however, real-time PCR is less expensive and more widely available in molecular labs. Improvements to DNA extraction yield and purity are crucial for real-time PCR assay optimization, but these improvements must be time- and cost-efficient. In this study, we improve upon previously published bulk DNA extraction methods by reducing bench time and costly materials. Our results indicate that the addition of caffeine and RNase A improves DNA extraction, resulting in higher amounts of target DNA in real-time PCR. Such improvements will enable the use of high throughput screening methods across multiple platforms to improve the probability of detection of Old World bollworm.

**Abstract:**

*Helicoverpa armigera* is among the most problematic agricultural pests worldwide due to its polyphagy and ability to evolve pesticide resistance. Molecular detection methods for *H. armigera* have been developed to track its spread, as such methods allow for rapid and accurate differentiation from the native sibling species *H. zea*. Droplet digital PCR (ddPCR) is a preferred method for bulk screening due to its accuracy and tolerance to PCR inhibitors; however, real-time PCR is less expensive and more widely available in molecular labs. Improvements to DNA extraction yield, purity, and throughput are crucial for real-time PCR assay optimization. Bulk DNA extractions have recently been improved to where real-time PCR sensitivity can equal that of ddPCR, but these new methods require significant time and specialized equipment. In this study, we improve upon previously published bulk DNA extraction methods by reducing bench time and materials. Our results indicate that the addition of caffeine and RNase A improves DNA extraction, resulting in lower Cq values during real-time PCR while reducing the processing time and cost per specimen. Such improvements will enable the use of high throughput screening methods across multiple platforms to improve the probability of detection of *H. armigera*.

## 1. Introduction

*Helicoverpa armigera* (Hübner) (Lepidoptera: Noctuidae), commonly referred to as Old World bollworm, is a major crop pest. Native to Europe, Asia, Africa, and Australia, it was once confined to the Eastern Hemisphere. In 2013, *H. armigera* was reported in Brazil [1], but it arrived earlier and established between 2006 and 2008 [2,3] and spread throughout South America. There is concern that *H. armigera* could become established in North America as well, based on its migratory ability and wide host range [4]. The first record of *H. armigera* in Puerto Rico occurred in 2014 [5] and the species has since become established there. In 2015, *H. armigera* was detected in Florida but due to management and subsequent monitoring is considered eradicated [5]. Larvae of *H. armigera* are highly polyphagous and have been recorded on 68 plant families worldwide [6]. These host plants include economically important crops like maize, tomato, soybean, cotton, and many specialty crops [7]. *H. armigera* has rapidly evolved resistance to many classes of pesticides and insecticidal proteins, making management increasingly difficult [6,8].

In the western hemisphere, *H. zea* (Boddie), the sister species of *H. armigera*, is an insect pest of major economic importance. *Helicoverpa zea* has independently evolved resistance to pesticides as well as *Bt* via strong selection pressure from widespread use [9,10]. The introduction of *H. armigera* to North America poses unique challenges because the two species are morphologically similar, requiring genitalic dissection to differentiate them, and have been shown to hybridize in both laboratory and natural settings [8]. Because dissection of individual moths is both difficult and time-consuming, molecular methods provide the most efficient means to identify *H. armigera*. In recent years, several molecular identification methods have been developed for detecting *H. armigera* both from interceptions and in surveys (e.g., [11,12,13,14,15]).

Continual large-scale screening for *H. armigera* is essential for management where recent introductions have been noted or the risk of introduction is high. Currently, the preferred method for molecular screening of bulk trap samples from domestic *H. armigera* surveys is using the droplet digital PCR methods described by Zink et al. [13] following bulk DNA extractions of 25–50 individual moths per reaction. This sample size allows for the identification of *H. armigera* regardless of sample quality and is efficient for additional screening of individual specimens in the case of a positive ddPCR result, which is carried out using real-time PCR following Gilligan et al. [11].

The benefits of screening samples with ddPCR are accuracy and sensitivity as well as high tolerance to the presence of PCR inhibitors, all of which are due to the droplet partitioning resulting in small reaction volumes [13,16,17,18]. Nevertheless, ddPCR is not widely available for insect screening due to the initial equipment expense and technical expertise required for meaningful implementation. Alternatively, real-time PCR is widely available and simple to implement and validate across multiple labs [19,20]. Real-time PCR is a sensitive and accurate molecular method with potential for use in screening individual specimens and bulk survey samples for *H. armigera* [11,14,21], but a bulk screening assay has not yet been developed for widespread use. The utility of real-time PCR for accurate detection of a single specimen in a bulk sample depends on developing a time and cost-efficient DNA extraction method of sufficient quality to minimize PCR inhibitors. Oliveira et al. [14] optimized a squish buffer [22] bulk extraction method by increasing the NaCl concentration in the buffer, determining the ideal centrifugation speed, and adding a post-centrifugation purification step using paramagnetic beads. Despite the clear benefits of reducing false negatives and significantly increasing end Relative Fluorescent Units (RFUs), bead purification increases sample handling, is more costly and time-consuming, and requires specialized equipment and training, all of which will complicate validation and inhibit widespread implementation of this protocol.

Here, we modify a bulk DNA extraction method by simplifying the protocol to minimize bench time and maximize extraction efficiency with the goal of equalizing our method with the real-time PCR and ddPCR efficiency of Oliveira et al. [14] using the real-time PCR assay as designed and optimized therein. This assay targets the 18S ribosomal DNA (rDNA) and the internal transcribed spacer 1 (ITS1) region to differentiate *H. armigera* from *H. zea*. The non-coding ITS regions of the rDNA array are highly effective for molecular species identification because the sequences are generally conserved within species with a high amount of variability between species and occur in high copy numbers in the genome [11,23,24]. Using the DNA extraction method detailed here, the bulk DNA extraction portion of the real-time PCR assay created by Oliveira et al. [14] is simplified so that *H. armigera* screening efforts can be accessible to and comparable across multiple labs.

## 2. Materials and Methods

### 2.1. Insect Specimens

The *H. zea* specimens used in this study were collected in the summer of 2022 in Larimer County, Colorado, USA using UNI-Trap Multi-Color bucket traps (Alpha Scents, Inc., Canby, OR, USA) containing the two-component *H. armigera* pheromone lure formulated and produced by USDA-APHIS Forest Pest Methods Laboratory (FPML) in Buzzards Bay, MA, USA. Bycatch was removed and *H. zea* months were visually identified and stored at 4 °C in paper envelopes until use. The *H. armigera* specimens used in this study were obtained from lab colonies (under growth conditions 25 ± 2 °C, 70 ± 5% RH, L:D 15:9) at the USDA-APHIS-FPML. Individuals collected from the colony were placed in 100% ethanol and frozen at −80 °C until use.

### 2.2. Bulk DNA Extraction

One dry *H. armigera* leg was added to varying numbers (10–100) of dry *H. zea* legs in a 1.5 mL microcentrifuge tube along with 1 mm zirconia/silica beads (Biospec Products, Bartlesville, OK, USA). Tubes were loaded into a mini-beadbeater (Biospec Products), and samples were pulverized for 1 min at high speed. The DNA extraction protocol outlined by Oliveira et al. [14] was used on all samples with adjustments to accommodate for the modifications when necessary (see Table 1). After the samples were pulverized, 15 µL per leg of squish buffer was added to each tube. Tubes were incubated overnight at 56 °C with agitation at 500 rpm in a dry bath Thermomixer FP (Eppendorf AG, Hamburg, Germany). After incubation, tubes were centrifuged at 8609× *g* for 10 min to pellet debris in an Eppendorf 5424 bench-top microcentrifuge. Select samples were bead-purified after DNA extraction using AMPure XP paramagnetic beads (Beckman Coulter, Inc., Brea, CA, USA), following the workflow described in Oliveira et al. [14].

### 2.3. Squish Buffer Formulation and Modifications

We used the optimized squish buffer formulation from Oliveira et al. [14] (hereafter referred to as the “O-buffer”), which consisted of 5 mM EDTA, 125 mM of NaCl, and 10 mM Tris, as our base formula and in control treatments. The O-buffer formula was modified from Perera et al. [12] and Gloor et al. [22]. A series of modifications to the O-buffer method were tested by following the bulk DNA extraction general protocol described above. Our modifications included the addition of freeze–thaw steps, the use of enzymes (Proteinase K, RNase A), the addition of different chemicals such as ascorbic acid, caffeine, and various surfactants, and the substitution of NaCl for KCl (Table 1).

### 2.4. Real-Time PCR

All DNA extraction methods were tested using the optimized real-time PCR assay outlined by Oliveira et al. [14]. The reactions consisted of 2× iTaq Universal Probes Supermix (Bio-Rad Laboratories Inc., Hercules, CA, USA) with 500 nM of each of the diagnostic primers and 160 nM of the diagnostic probe (Table 2), 400 nM of each of the control primers and 400 nM of the control probe (Table 2), 1 µL DNA of varying concentration, and water to complete the dilution of the Supermix. All real-time PCR was carried out using a Bio-Rad CFX96 Real-Time PCR system (Bio-Rad Laboratories Inc.) using the following conditions: 95 °C for 3 min, followed by 40 cycles of 95 °C for 15 s, then 1 min at 56 °C, with data capture at the end of the amplification step. A lid temperature of 105 °C was maintained throughout all cycles. The CFX Manager v 3.1 software (Bio-Rad Laboratories Inc.) was used to visualize and initially analyze data. The baseline threshold was set to 100 to match that of Oliveira et al. [14].

### 2.5. DNA Extraction Modification Testing

After testing each squish buffer modification (Table 1) against the O-buffer, the best-performing modifications were combined into one protocol, containing RNase A and caffeine (RC-buffer), described in Table 3. We tested the RC-buffer against each of the best-performing modifications alone (RNase A and Caffeine) and against the O-buffer. We also tested the RC-buffer at ratios of one *H. armigera* leg to increasing numbers of *H. zea* legs (up to 100) to determine positive detection in larger sample sizes. To simulate real-world sample size and variation, we tested 70 biological replicates of the RC-buffer extraction method using a ratio of one *H. armigera* leg to 50 *H. zea* legs and compared them to positive controls of the same target-to-non-target ratio consisting of O-buffer extraction with and without bead purification. We ran three technical replicates for each sample on real-time PCR.

### 2.6. Statistical Analysis

The quantification cycle (Cq) and end RFU values of each of the 70 biological replicates of RC-buffer as well as the three biological replicates of the O-buffer treatments with and without bead purification (BP) were averaged for analysis. A one-way Analysis of Variance (ANOVA) was carried out in R version 4.3.2 [26] to evaluate the statistical significance of real-time PCR Cq values as well as end RFU values for both probes between treatments across the three technical replicates. When deemed significant, post hoc comparisons were made using the Tukey HSD test in the R agricolae package [27].

### 2.7. ddPCR

For ddPCR, we followed the EvaGreen assay protocol used in Oliveira et al. [14]. This included 10 µL 2× EvaGreen ddPCR Supermix (Bio-Rad Laboratories Inc.), 200 nM of each primer (Table 2), 1 µL of DNA that was fragmented in QIAshredder columns (Qiagen, Hilden, Germany) and diluted 1:50, and 8 µL water. The thermocycler program was as follows: 95 °C for 5 min followed by 40 cycles of 95 °C for 30 s, 56 °C for 30 s, and 72 °C for 1 min, 4 °C for 5 min, 90 °C for 5 min, and an infinite hold at 4 °C. All PCR was carried out in a C1000 Touch thermocycler with a deep-well reaction module (Bio-Rad Laboratories Inc.) using a ramp rate of 2 °C/s between all steps and a constant lid temperature of 105 °C. All reactions were run on the QX200 Droplet Digital PCR System (Bio-Rad Laboratories Inc.) and QuantaSoft v1.7.4.0917 (Bio-Rad Laboratories Inc.) software was used for analyses. The threshold for calling intermediate droplets positive was determined using the program ‘definetherain’ (definetherain.org.uk; [28]).

## 3. Results

### 3.1. Performance of DNA Extraction Modifications

Modifications to the O-buffer extraction method were tested using a ratio of 1 *H. armigera* leg to 10 *H. zea* legs in four extraction groups, using an unmodified O-buffer extraction of the same leg ratio without bead purification (BP) as the positive control replicated for each group (Table 4). The addition of RNase A and caffeine each produced lower Cq values compared to the positive control for the *H. armigera* diagnostic probe (Table 4). The RNase A modification resulted in a Cq decrease of 1.22 cycles for the *H. armigera* probe, while the caffeine modification resulted in a Cq decrease of 1.85 cycles (Table 4). For the control probe, RNase A produced a Cq that was 0.51 cycles higher than the positive control and caffeine produced a Cq that was 0.05 cycles higher than the positive control. The addition of ascorbic acid, CTAB, and SDS produced false negative results (no Cq value) for the *H. armigera* probe (Table 4). Adding freeze–thaw cycles, Proteinase K, Triton X, Tween 20, and substituting KCl for NaCl each resulted in higher Cq values for the *H. armigera* probe than the positive control (Table 4).

### 3.2. RNase A and Caffeine Improve Squish Buffer Extraction

When both RNase A and caffeine were combined in a single extraction method (RC-buffer) to test for an additive effect, the RC-buffer produced an average Cq value of 22.92 for the *H. armigera* probe, lower than either of the modifications alone. Overall, extractions that contained RNase A, caffeine, or both produced lower Cq values and higher end RFUs for the *H. armigera* probe than the positive control (O-buffer without BP; Table 5). The RC-buffer extraction method resulted in a decrease of the Cq value between treatment and control by 1.05 cycles compared to caffeine alone and by 0.93 cycles compared to RNase A alone.

### 3.3. Real-Time PCR Bulk Samples Limit of Detection

To test the utility of the RC-buffer extraction method in larger sample sizes similar to actual bulk testing, experimental ratios of one *H. armigera* leg to 10, 25, 50, 75, and 100 *H. zea* legs were made and tested using this protocol. At the largest sample size of one *H. armigera* leg to 100 *H. zea* legs, there was positive detection of the *H. armigera* DNA in the sample with a Cq value of 21.75 for the diagnostic probe. For all ratios tested, the Cq values for the diagnostic probe ranged from 19.09 to 21.75, while the Cq values for the control probe ranged from 14.14 to 15.15. End RFU ranged from 8871.71 to 11,573 for the diagnostic probe and from 11,391.77 to 12,093.79 for the control probe (Table 6).

### 3.4. RC-Buffer Performance and Repeatability

**Figure 1:**
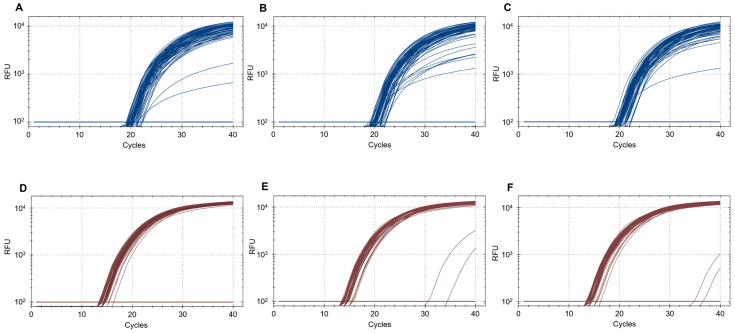
Real-time PCR amplification curves representing 70 samples of the RC-buffer extraction and three positive control samples of the O-buffer extraction with and without BP using a ratio of one *H. armigera* to 50 *H. zea* legs, two negative control samples of RC-buffer with 50 *H. zea* legs and no *H. armigera* legs, and two NTC samples with water instead of DNA. (**A**–**C**) show amplification curves for the first, second, and third technical replicates for the *H. armigera* probe. (**D**–**F**) show amplification curves for three technical replicates for the *H. armigera* probe.

We tested the RC-buffer extraction method against the O-buffer extraction with and without BP using leg ratios of one *H. armigera* leg to 50 *H. zea* legs. Out of 70 biological replicates of the RC-buffer extraction tested, there were no false negatives (Table 7, Figure 1, Supplementary Table S1). There was a significant difference in the *H. armigera* probe Cq values between treatments (*p* < 0.001; Table 8). Pairwise comparisons indicated that the RC-buffer produced significantly lower Cq values than the O-buffer treatment with and without BP (*p* < 0.001; Table 9). The unpurified positive control Cq was significantly lower than the positive control with BP (*p* = 0.04; Table 9). Following the same pattern as the *H. armigera* probe, the control probe had Cq values that differed significantly between treatments (*p* < 0.001; Supplementary Table S2) and pairwise comparisons indicated that the RC-buffer produced significantly lower Cq values than all other treatments while the positive control without BP produced significantly lower Cq values than the positive control with BP (*p* < 0.001; Supplementary Table S3).

The end RFU for the *H. armigera* probe significantly differed among treatments (*p* = 0.004; Supplementary Table S4). The O-buffer without BP produced the lowest end RFU at 5455 and the O-buffer with BP produced the highest end RFU at 10,483, while the RC-buffer averaged between 8441 and 8595 (Table 7). Pairwise comparisons between the treatments for the *H. armigera* probe indicated that the O-buffer with BP and the RC-buffer extraction RFU did not significantly differ from one another (Supplementary Table S5). The RC-buffer had a significantly higher end RFU than the O-buffer without BP (*p* = 0.03) and the O-buffer with BP had a significantly higher end RFU than without BP (*p* = 0.003; Supplementary Table S5). For the control probe, the end RFU remained consistent between all treatments and showed no significant differences, with values ranging from 12,003 to 12,691 (*p* = 0.91; Supplementary Table S6).

### 3.5. ddPCR with RC-Buffer

**Figure 2:**
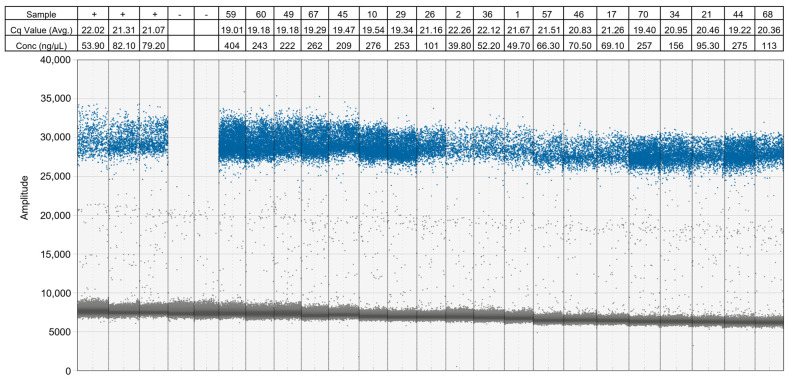
Results from the ddPCR assay using EvaGreen to detect *H. armigera* in a subset of RC-buffer extractions. Each number-labeled sample contained one *H. armigera* leg and 50 *H. zea* legs. Sample names and Cq values (averaged from three technical replicates and rounded to two decimal points) correspond to Supplementary Table S1. Samples labeled “+” are positive controls consisting of O-buffer extractions with one *H. armigera* leg and 50 *H. zea* legs. Samples labeled “-” are negative controls consisting of O-buffer extractions with 50 *H. zea* legs and no *H. armigera* legs. Positive droplets are blue and negative droplets are grey. Conc (ng/uL) is the calculated starting target DNA concentration.

To confirm compatibility between the new buffer formulation and ddPCR chemistry we tested a subset of the one *H. armigera* leg to 50 *H. zea* legs ratio RC-buffer extractions on ddPCR using the optimized EvaGreen assay described in Oliveira et al. [14]. The samples with the highest and lowest Cq and RFU values from the real-time PCR results were tested with the ddPCR assay. When run on real-time PCR, these samples produced average Cq values ranging from 19.01 ± 0.18 to 22.26 ± 0.04 (see Supplementary Table S1). Each of the samples run on ddPCR was positive for *H. armigera* (Figure 2). The samples with the lowest Cq values on real-time PCR correspond to the samples with the highest numbers of positive droplets in ddPCR (depicted as the darkest blue and most dense clusters of blue positive droplets in Figure 2).

## 4. Discussion

Due to the threat the introduction of *H. armigera* to the U.S. poses to food security, a rapid diagnostic method is critical and must be widely adopted to track its spread. Currently, ddPCR is used for bulk screening due to its accuracy and tolerance to PCR inhibitors; however, real-time PCR is a more common, accessible, and less expensive technology that can be adapted to bulk sample screening for *H. armigera*. The newly optimized DNA bulk extraction method presented here includes the addition of caffeine and RNase A to a single-step extraction technique that significantly improves upon recently published methods by increasing extraction efficiency and reducing processing time and cost per specimen for use with real-time PCR.

The squish buffer DNA extraction method has been shown to be successful for ddPCR [13] and was used previously to screen bulk samples by high-resolution melt (HRM; [12]). Using HRM, Perera et al. [12] were able to successfully screen samples of one *H. armigera* in 24 *H. zea* but results were inconsistent and not easily replicated in other labs, with different equipment, or with different reagents, making validation of this protocol difficult (see [13]). To develop an effective screening method based on real-time PCR, Oliveira et al. [14] designed a new assay based on the same locus (ITS1) as Perera et al. [12] and modified the squish buffer method by increasing the concentration of NaCl, using a slower centrifugation speed, and adding a bead purification step. Combined, these changes led to the consistent detection of a single *H. armigera* leg among 50 *H. zea* legs, a ratio consistent with what is typically used for bulk extractions from survey samples. Bead purification significantly increased detection in bulk samples, but also increased processing time, sample handling, and cost per sample. In order to eliminate the bead purification and increase assay simplicity and speed, in this study we further modified the squish buffer formulation with the goal of increasing bulk DNA extraction efficiency for improved target detection. We found that adding freeze–thaw steps, Proteinase K, Triton X, Tween 20, and substitution of NaCl for KCl did not improve extraction efficiency as measured by Cq value when compared to the control samples. Proteinase K is effective for removing proteins but will act as an inhibitor by interfering with Taq polymerase during PCR if it is not effectively removed from extracted DNA [29,30]. The addition of ascorbic acid, CTAB, and SDS produced false negative results (no Cq value) for the three samples (Table 4), likely because both CTAB and SDS can delay amplification and inhibit PCR even at low concentrations [31]. Because our goal was to simplify the extraction, we chose not to introduce additional purification steps to remove surfactants or enzymes. The addition of RNase A and caffeine each improved bulk DNA extraction by producing lower Cq values than the O-buffer extraction.

Co-extraction of RNA in DNA extraction protocols leads to a less pure DNA product and the RNA becomes a contaminant in spectrofluorometry, leading to problems when quantifying DNA concentration [32]. In our case, where PCR is the end goal, RNA remaining in the DNA extract can bind to template DNA and inhibit amplification in PCR [33]. By adding RNase A to the squish buffer, RNA impurities are removed without introducing PCR inhibitors. Caffeine has been shown to slow protein aggregation [34], making DNA more available for extraction. Tsabar et al. [35] showed that caffeine treatment impairs DNA repair and degrades restriction enzymes. As such, caffeine’s ability to degrade restriction enzymes may be one reason why it is effective in our bulk DNA extraction by blocking proteins that would otherwise interfere with template DNA and possibly Taq, thereby inhibiting PCR. Combining caffeine and RNase A in the squish buffer extraction produced significantly lower Cq values than the O-buffer with and without BP for the diagnostic *H. armigera* probe, decreased processing time and sample handling compared to bead purification, did not require special equipment, and added only minimal bench time (approximately 45 min more than O-buffer without BP).

Along with increased salt concentration and secondary bead purification, Oliveira et al. [14] found that running the diagnostic assay without the control probe significantly increased the accuracy and precision of their real-time PCR results both with and without bead purification. We rejected the idea of running the assay without the control probe despite improvements to the DNA extraction process because it is necessary to ensure that PCR is occurring in each well. The RC-buffer extraction results detailed here consistently matched the efficiency of Oliveira et al. [14] real-time PCR results with no control probe when run as a duplex real-time PCR assay.

The RC-buffer extraction is highly repeatable and was tested with 70 biological replicates at a ratio of one *H. armigera* leg to 50 *H. zea* legs to represent a realistic bulk sample size, which was run with three technical replicates each. This sample size was chosen because the concentration of *H. armigera* DNA relative to *H. zea* would be sufficient in positive samples to reduce false negatives due to low DNA quality or concentration. If a positive is found, each specimen must be extracted individually and screened using real-time PCR with the protocol from Gilligan et al. [11] to confirm the total number of *H. armigera* present in the sample and then further confirmed by CO1 barcoding and possibly dissection for regulatory purposes. Because each of the 70 replicates contains legs from different *H. armigera* individuals, variation in the *H. armigera* probe results is expected due to slight differences in leg size and quality resulting in differences in target (*H. armigera*) DNA concentrations relative to non-target (*H. zea*) DNA. Such variation is expected in real-world trap samples as well and needs to be accounted for in the interpretation of results. To determine if a sample is positive, we developed the following interpretation guidelines based on the Cq values of the 70 samples extracted with RC-buffer with the ratio of one *H. armigera* leg to 50 *H. zea* legs and based on findings from Oliveira et al. [14]. With a baseline threshold of 100 RFU for each probe, the control probe Cq must be >5 and ≤31 to ensure the presence of lepidopteran DNA, while the diagnostic probe Cq value must be >10 and ≤37. The difference between the control probe and the diagnostic probe must be >0 and ≤8. Additionally, all samples tested on ddPCR were positive for *H. armigera* (Figure 2). The correlation between positive results in our samples for both ddPCR and real-time PCR confirms the conclusions of Oliveira et al. [14] that this real-time PCR assay equalizes the success rate of ddPCR in detecting *H. armigera* from bulk samples of up to 1:100 individuals.

The findings from our study are important because successful mass screening efforts for *H. armigera* depend on a rapid and efficient bulk DNA extraction method that produces DNA of sufficient quality to be used for real-time PCR. This process needs to be repeatable and accessible to promote use across multiple labs. The addition of RNase A and caffeine in this modified squish buffer DNA extraction results in a DNA extraction of sufficient quality for use in detecting *H. armigera* in bulk samples using real-time PCR without adding bench time or specialized methods so it can be widely implemented across labs. The findings presented here regarding the addition of RNase A and caffeine might also be applicable to similar bulk extractions of other arthropod species.

## Figures and Tables

**Table 1:** Descriptions of each of the extraction modifications tested.

Modification	Description
**Freeze–thaw**	After adding O-buffer, samples were placed in a −80 °C freezer for 15 min. Samples were thawed completely to room temp on the bench (15 min). Each sample was subjected to three freeze–thaw cycles before the overnight incubation step in the DNA extraction protocol.
**RNase A**	After overnight incubation, samples were cooled to 37 °C and 1 µL of 5 µg/µL RNase A was added to each tube. Samples were incubated at 37 °C for 30 min before the centrifugation step of the DNA extraction protocol.
**Proteinase K**	A total of 1 µL of 5 µg/µL Proteinase K was diluted into 300 µL of O-buffer before adding the appropriate amount of buffer to each tube (15 µL/leg). Samples were incubated overnight at 65 °C shaking at 500 rpm in the dry bath before the centrifugation step of the DNA extraction protocol.
**Ascorbic Acid**	Ascorbic acid powder was added to the O-buffer to make a 1% (*w*/*v*) solution and the DNA extraction protocol was carried out as usual.
**Caffeine**	Caffeine (99.7%) powder was added to the O-buffer to make a 1% (*w*/*v*) solution and the DNA extraction protocol was carried out as usual.
**Surfactants**	**Tween 20** was added to the O-buffer to make a 1% (*v*/*v*) solution and the DNA extraction protocol was carried out as usual.
	**SDS** powder was added to the O-buffer to make a 1% (*w*/*v*) solution and the DNA extraction protocol was carried out as usual.
	**Triton X** was added to the O-buffer to make a 1% (*v*/*v*) solution and the DNA extraction protocol was carried out as usual.
	**CTAB** powder was added to O-buffer to make a 1% (*w*/*v*) solution and the DNA extraction protocol was carried out as usual.
**Potassium Chloride**	A total of 125 mM NaCl was substituted for 125 mM KCl in the O-buffer prior to following the DNA extraction protocol. Samples were incubated overnight at 65 °C shaking at 500 rpm in the dry bath before the centrifugation step.

**Table 2:** Diagnostic and control primers and probes used in this study.

Primer Name	Use	Sequence	Tm (°C)	Source
Harm_18S_1944F	Diagnostic	5′-AACGTAAACAATAATCCACACACCA	55.1	[14]
Harm_18S_2154R	Diagnostic	5′-CGCGGATTTTTGTGTTTTGTGT	56.5	[14]
Harm_18S_1969P	Diagnostic	5′-6-FAM-CTAGAGGAC-ZEN-ACAGAGTCGAACG-IowaBlackFQ	56.4	[14]
RT-18S-F2	Control	5′-ACCGCCCTAGTTCTAACCGTAAA	57.8	[25]
RT-18S-R2	Control	5′-CCGCCGAGCCATTGTAGTAA	57.3	[25]
RT-18S-P2	Control	5′-Quasar670-TGTCATCTAGCGATCCGCCGA-BHQ-2	60.3	[25]

**Table 3:** Description of the RC-buffer modification.

Modification	Description
**RNase A and Caffeine (RC-buffer)**	Caffeine (99.7%) powder was added to the O-buffer to make a 1% (*w*/*v*) solution in which samples were incubated at 56 °C shaking at 500 rpm overnight. Samples were cooled to 37 °C and 1 µL of 5 µg/µL RNase A was added to each tube. Samples were then incubated at 37 °C for 30 min before centrifugation.

**Table 4:** Real-time PCR results for extraction modifications. Ratios of one *H. armigera* leg to 10 *H. zea* legs were used for all modifications and positive controls. Negative controls contained 10 *H. zea* legs and no *H. armigera* legs.

Extraction Group 1	*H. armigera* Probe Cq	Ctrl Probe Cq	*H. armigera* Probe End RFU
Freeze–thaw	23.10	14.69	6694.90
Pos Ctrl 1 (O-buffer)	22.01	14.89	5266.14
Neg Ctrl freeze–thaw	N/A	14.85	0.11
Neg Ctrl 1 (O-buffer)	N/A	17.35	−3.54
**Extraction Group 2**	***H. armigera*** **probe Cq**	**Ctrl probe Cq**	***H. armigera*** **probe end RFU**
Proteinase K	25.19	15.89	5589.05
Proteinase K + RNase A	26.86	16.61	3898.19
RNase A	21.85	15.67	10,411.23
Pos Ctrl 2 (O-buffer)	23.07	15.16	4485.38
Neg Ctrl Proteinase K	N/A	16.03	10.70
Neg Ctrl Proteinase K + RNase A	N/A	16.60	15.60
Neg Ctrl RNase A	N/A	15.70	9.35
Neg Ctrl 2 (O-buffer)	N/A	15.92	4.55
**Extraction Group 3**	***H. armigera*** **probe Cq**	**Ctrl probe Cq**	***H. armigera*** **probe end RFU**
Caffeine	20.85	15.28	11,272.38
CTAB	N/A	N/A	7.81
SDS	N/A	N/A	2.45
Triton X	26.14	15.12	5033.07
Tween 20	32.06	23.33	883.77
Ascorbic Acid	N/A	29.85	−24.66
Pos Ctrl 3 (O-buffer)	22.70	15.23	9577.66
Neg Ctrl Caffeine	N/A	14.83	−13.20
Neg Ctrl CTAB	N/A	N/A	90.40
Neg Ctrl SDS	N/A	N/A	1.62
Neg Ctrl Triton X	N/A	15.70	−21.00
Neg Ctrl Tween 20	N/A	18.23	−33.40
Neg Ctrl Ascorbic Acid	19.26	29.85	274.00
Neg Ctrl 3 (O-buffer)	N/A	15.84	−22.00
**Extraction Group 4**	***H. armigera*** **probe Cq**	**Ctrl probe Cq**	***H. armigera*** **probe end RFU**
Potassium Chloride	26.66	16.32	4379.00
Pos Ctrl 4 (O-buffer)	24.74	15.78	7178.00
Neg Ctrl Potassium Chloride	N/A	15.81	−19.90
Neg Ctrl 4 (O-buffer)	N/A	15.85	−24.40

**Table 5:** Real-time PCR results for caffeine and RNase extraction methods alone and for the RC-buffer. Ratios of one *H. armigera leg* to 10 *H. zea* legs were used in all samples except for negative controls which contained 10 *H. zea* legs and no *H. armigera* legs.

Treatment	*H. armigera* Probe Mean Cq	Ctrl Probe Mean Cq	*H. armigera* Probe Mean RFU	Biological Reps	Technical Reps
Caffeine	23.97	15.50	7882.92	2	2
RNase A	23.85	15.80	7104.61	2	2
RC-buffer	22.92	15.19	8641.85	2	2
Pos Ctrl O-buffer	26.01	16.31	3246.12	1	2
Neg Ctrl Caffeine	N/A	14.38	−16.10	1	2
Neg Ctrl RNase A	N/A	15.95	−28.30	1	2
Neg Ctrl O-buffer	N/A	17.61	−45.10	1	2

**Table 6:** Real-time PCR results for increasing ratios of *H. armigera* legs–*H. zea* legs using the RC-buffer extraction method.

Ratio	*H. armigera* Probe Cq	*H. armigera* Probe RFU	Ctrl Probe Cq	Ctrl Probe RFU
1:10	19.31	11,023.25	15.15	11,460.30
1:25	19.09	11,536.28	14.17	11,391.77
1:50	19.60	11,573.20	14.50	12,093.79
1:75	20.06	10,287.55	14.14	11,739.34
1:100	21.75	8871.71	15.06	11,650.65
0:50	N/A	−2.47	14.02	11,618.10

**Table 7:** Real-time PCR results for 70 replicates of the RC-buffer extraction and three replicates of positive control samples with and without BP. Ratios of one *H. armigera leg* to 50 *H. zea* legs were used in all samples, except for negative controls which contained 50 *H. zea* legs and no *H. armigera* legs.

Treatment	Technical Rep	*H. Armigera* Probe (Mean Cq ± SD)	*H. Armigera* Probe (Mean RFU ± SD)	Ctrl Probe (Mean Cq ± SD)	Ctrl Probe (Mean RFU ± SD)	N
RC-buffer	1	20.20 ± 0.64	8571.30 ± 2066.17	14.13 ± 0.38	12,556.70 ± 516.48	70
	2	20.20 ± 0.69	8440.59 ± 2232.47	14.12 ± 0.42	12,063.06 ± 556.49	70
	3	20.22 ± 0.66	8595.29 ± 183.37	14.09 ± 0.35	12,294.99 ± 567.51	70
O-buffer	1	21.49 ± 0.37	7139.84 ± 1047.40	14.70 ± 0.09	12,512.89 ± 429.74	3
	2	21.39 ± 0.66	5455.43 ± 1997.88	14.64 ± 0.23	12,003.15 ± 223.29	3
	3	21.52 ± 0.50	7038.41 ± 1655.11	14.70 ± 0.15	12,406.09 ± 160.18	3
O-buffer BP	1	21.54 ± 0.57	10,482.84 ± 834.94	15.69 ± 0.53	12,691.07 ± 420.73	3
	2	21.74 ± 0.50	8924.51 ± 1367.38	15.70 ± 0.14	12,123.06 ± 200.55	3
	3	21.77 ± 0.45	9913.33 ± 1685.27	15.85 ± 0.40	12,358.21 ± 352.76	3
Neg Ctrl RC-buffer	1		0.56	13.87	13,119.80	1
	2		2.25	13.88	12,577.48	1
	3		3.71	13.95	12,972.91	1
Neg Ctrl O-buffer	1		0.70	13.67	13,191.33	1
	2		1.91	13.80	12,439.15	1
	3		1.80	13.84	13,193.89	1

**Table 8:** Analysis of variance results comparing *H. armigera* probe Cq values across treatments (RC-buffer, O-buffer, and O-buffer BP).

	Df	Sum Sq	Mean Sq	F Value	Pr (>F)
**Treatment**	2.00	3.81	1.91	284.50	1.14 × 10^−6^ ***
**Residuals**	6.00	0.04	0.01		

*** indicates *p* < 0.0001.

**Table 9:** Tukey HSD test results comparing *H. armigera* probe Cq values across treatments.

Treatment	Mean Difference	Std Error	*p*-Value	95% CI	
				Lower Bound	Upper Bound
O-buffer–O-buffer BP	0.2183386	0.0472508	0.0392132	0.01330797	0.4233691
RC-buffer–O-buffer	−1.2582476	0.0472508	0.0000033	−1.46327816	−1.053217
RC-buffer–O-buffer BP	−1.4765861	0.0472508	0.0000014	−1.68161671	−1.2715555

## Data Availability

All data generated and analyzed for this study are included in this published article and the associated Supplementary Information Files. Additional inquiries can be directed to the corresponding author.

## Supplementary Materials

The following supporting information can be downloaded at: https://www.mdpi.com/article/10.3390/insects15080585/s1, Table S1: Real-time PCR results for 70 replicates of leg ratios; Table S2: Analysis of Variance for control probe Cq; Table S3: Tukey HSD test results for control probe Cq; Table S4: Analysis of Variance for diagnostic probe end RFU; Table S5: Tukey HSD test results for diagnostic probe end RFU; Table S6: Analysis of Variance table for control probe end RFU.

## References

[1] Czepak C., Albernaz K.C., Vivan L.M., Guimaraes H.O., Carvalhais T. (2013). First reported occurrence of *Helicoverpa armigera* (Hubner) (Lepidoptera: Noctuidae) in Brazil. Pesqui. Agropecu. Trop..

[2] Tay W.T., Soria M.F., Walsh T., Thomazoni D., Silvie P., Behere G.T., Anderson C., Downes S. (2013). A brave new world for an old world pest: *Helicoverpa armigera* (Lepidoptera: Noctuidae) in Brazil. PLoS ONE.

[3] Sosa-Gomez D.R., Specht A., Paula-Moraes S.V., Lopes-Lima A., Yano S.A.C., Micheli A., Morais E.G.F., Gallo P., Pereira P.R.V.S., Salvadori J.R. (2016). Timeline and geographical distribution of *Helicoverpa armigera* (Hubner) (Lepidoptera, Noctuidae: Heliothinae) in Brazil. Rev. Bras. Entomol..

[4] Kriticos D.J., Ota N., Hutchison W.D., Beddow J., Walsh T., Tay W.T., Borchert D.M., Paula-Moraes S.V., Czepak C., Zalucki M.P. (2015). The potential distribution of invading *Helicoverpa armigera* in North America: Is it just a matter of time?. PLoS ONE.

[5] Tembrock L.R., Timm A.E., Zink F.A., Gilligan T.M. (2019). Phylogeography of the recent expansion of *Helicoverpa armigera* (Lepidoptera: Noctuidae) in South America and the Caribbean basin. Ann. Entomol. Soc. Am..

[6] Cunningham J.P., Zalucki M.P. (2014). Understanding heliothine (Lepidoptera: Heliothinae) pests: What is a host plant?. J. Econ. Entomol..

[7] Pomari-Fernandes A., Bueno A.d.F., Sosa-Gomez D.R. (2015). *Helicoverpa armigera*: Current status and future perspectives in Brazil. Curr. Agric. Sci. Technol..

[8] Anderson C.J., Oakeshott J.G., Tay W.T., Gordon K.H.J., Zwick A., Walsh T.K. (2018). Hybridization and gene flow in the mega-pest lineage of moth, Helicoverpa. Proc. Natl. Acad. Sci. USA.

[9] Stahlke A.R., Chang J., Tembrock L.R., Sim S.B., Chudalayandi S., Geib S.M., Scheffler B.E., Perera O.P., Gilligan T.M., Childers A.K. (2023). A chromosome-scale genome assembly of a *Helicoverpa zea* strain resistant to *Bacillus thuringiensis* Cry1Ac insecticidal protein. Genome Biol. Evol..

[10] North H.L., Fu Z., Metz R., Stull M.A., Johnson C.D., Shirley X., Crumley K., Reisig D., Kerns D.L., Gilligan T.M. (2024). Rapid adaptation and interspecific introgression in the North American crop pest *Helicoverpa zea*. Mol. Biol. Evol..

[11] Gilligan T.M., Tembrock L.R., Farris R.E., Barr N.B., van der Straten M.J., van de Vossenberg B.T.L.H., Metz-Verschure E. (2015). A multiplex real-time PCR assay to diagnose and separate *Helicoverpa armigera* and *H. zea* (Lepidoptera: Noctuidae) in the New World. PLoS ONE.

[12] Perera O.P., Allen K.C., Jain D., Purcell M., Little N.S., Luttrell R.G. (2015). Rapid identification of *Helicoverpa armigera* and *Helicoverpa zea* (Lepidoptera: Noctuidae) using ribosomal RNA internal transcribed spacer 1. J. Insect Sci..

[13] Zink F.A., Tembrock L.R., Timm A.E., Farris R.E., Perera O.P., Gilligan T.M. (2017). A droplet digital PCR (ddPCR) assay to detect *Helicoverpa armigera* (Lepidoptera: Noctuidae) in bulk trap samples. PLoS ONE.

[14] Oliveira T.M.R., Zink F.A., Menezes R.C., Dianese E.C., Albernaz-Godinho K.C., Cunha M.G., Timm A.E., Gilligan T.M., Tembrock L.R. (2021). Assay optimization can equalize the sensitivity of real-time PCR with ddPCR for detection of *Helicoverpa armigera* (Lepidoptera: Noctuidae) in bulk samples. Insects.

[15] Rich M., Noh E., Wang H., Greene J., Gilligan T., Reay-Jones F.P.F., Turnbull M., Zink F. (2023). Field-based recombinase polymerase amplification and lab-based qPCR assays for detection of *Helicoverpa armigera*. J. Econ. Entomol..

[16] Hindson B.J., Ness K.D., Masquelier D.A., Belgrader P., Heredia N.J., Makarewicz A.J., Bright I.J., Lucero M.Y., Hindessen A.L., Legler T.C. (2011). High-throughput droplet digital PCR system for absolute quantification of DNA copy number. Anal. Chem..

[17] Zink F.A., Tembrock L.R., Timm A.E., Gilligan T.M. (2022). A droplet digital PCR (ddPCR) assay to detect *Phthorimaea absoluta* (Lepidoptera: Gelechiidae) in bulk trap samples. J. Econ. Entomol..

[18] Hou Y., Chen S., Zheng Y., Zheng X., Lin J.-M. (2023). Droplet-based digital PCR (ddPCR) and its applications. Trends Anal. Chem..

[19] Bustin S.A., Benes V., Garson J.A., Hellemans J., Huggett J., Kubista M., Mueller R., Nolan T., Pfaffl M.W., Shipley G.L. (2009). The MIQE guidelines: Minimum information for publication of quantitative real-time PCR experiments. Clin. Chem..

[20] van de Vossenberg B.T.L.H., te Braak N., Timm A.E., van Noort T.H., Gilligan T.M. (2024). Development and validation of a real-time PCR for the molecular identification of *Thaumatotibia leucotreta* (Lepidoptera: Tortricidae: Olethreutinae) intercepted in trade. Phytofrontiers.

[21] Jarrett S., Morgan J.A.T., Wlodek B.M., Brown G.W., Urech R., Green P.E., Lew-Tabor A.E. (2010). Specific detection of the Old World screwworm fly, *Chrysomya bezziana*, in bulk fly trap catches using real-time PCR. Med. Vet. Entomol..

[22] Gloor G.B., Preston C.R., Johnson-Schlitz D.M., Nassif N.A., Phillis R.W., Benz W.K., Robertson H.M., Engles W.R. (1993). Type I repressors of P element mobility. Genetics.

[23] Coleman A.W. (2007). Pan-eukaryote ITS2 homologies revealed by RNA secondary structure. Nucleic Acids Res..

[24] Zink F.A., Tembrock L.R., Timm A.E., Gilligan T.M. (2023). Ultra-deep sequencing of 45S rDNA to discern intragenomic diversity in three *Chrysodeixis* species for molecular identification. Sci. Rep..

[25] Barr N.B., Ledezma L.A., Farris R.E., Epstein M.E., Gilligan T.M. (2011). A multiplex real-time polymerase chain reaction assay to diagnose *Epiphyas postvittana* (Lepidoptera: Tortricidae). J. Econ. Entomol..

[26] Team R.C. (2020). R: A language and Environment for Statistical Computing.

[27] Mendiburu F.D. Agricolae: Statistical Procedures from Agricultural Research, 1.3-7; R Package Version 1.3-7: 2023. https://CRAN.R-project.org/package=agricolae.

[28] Jones M., Williams J., Gartner K., Phillips R., Hurst J., Frater J. (2014). Low copy target detection by Droplet Digital PCR through application of a novel open access bioinformatic pipeline, ‘definetherain’. J. Virol. Methods.

[29] Abolmaaty A., El-Shemy M.G., Khallaf M.F., Levin R.E. (1998). Effect of lysing methods and their variables on the yield of *Escherichia coli* O157:H7 DNA and its PCR amplification. J. Microbiol. Methods.

[30] Schrader C., Schielke A., Ellerbroek L., Johne R. (2012). PCR inhibitors–occurrence, properties, and removal. J. Appl. Microbiol..

[31] Demeke T., Jenkins G.R. (2010). Influence of DNA extraction methods, PCR inhibitors and quantification methods on real-time PCR assay of biotechnology-derived traits. Anal. Bioanal. Chem..

[32] Sanchez I., Remm M., Frasquilho S., Betsou F., Mathieson W. (2015). How severely is DNA quantification hampered by RNA co-extraction?. Biopreserv. Biobank..

[33] Yuen P.S.T., Brooks K.M., Li Y. (2001). RNA: A method to specifically inhibit PCR amplification of known members of a multigene family by degenerate primers. Nucleic Acids Res..

[34] Hosoi T., Toyoda K., Nakatsu K., Ozawa K. (2014). Caffeine attenuated ER stress-induced leptin resistance in neurons. Neurosci. Lett..

[35] Tsabar M., Eapen V.V., Mason J.M., Memisoglu G., Waterman D.P., Long M.J., Bishop D.K., Haber J.E. (2015). Caffeine impairs resection during DNA break repair by reducing the levels of nucleases Sae2 and Dna2. Nucleic Acid. Res..

