# Supplementary material for: An Improved Bulk DNA Extraction Method for Detection of Helicoverpa armigera (Lepidoptera: Noctuidae) Using Real-Time PCR
Source: Insects. 2024 Aug 1;15(8):585. doi: 10.3390/insects15080585 (PMC11355060; doi:10.3390/insects15080585)
Supplement: Supplementary file 1 [file insects-15-00585-s001.zip › insects-3110775-supplementary.pdf]

Table S1. Real-time PCR results for 70 replicates of the RC buffer extraction vs. 3 replicates each of bead purified and non-bead purified positive control samples. Ratios of 1 *H. armigera leg* to 50 *H. zea* legs were used in all samples.

	***H. armigera* probe**								**Control probe**						
	**Technical Rep. 1**		**Technical Rep. 2**		**Technical Rep. 3**				**Technical Rep. 1**		**Technical Rep. 2**		**Technical Rep. 3**			
Sample	Cq	End RFU	Cq	End RFU	Cq	End RFU	SD Cq	SD End RFU	Cq	End RFU	Cq	End RFU	Cq	End RFU	SD Cq	SD End RFU
1	21.47	6749.94	22.16	7629.64	21.38	7844.07	0.35	473.40	14.45	11899.34	14.87	11669.44	14.34	11740.95	0.23	96.06
2	22.24	1522.28	22.22	2321.74	22.31	6787.38	0.04	2316.66	15.44	10951.82	15.46	10257.03	15.38	11380.72	0.04	463.01
3	20.37	10325.26	20.24	8873.93	20.32	8790.16	0.05	704.74	14.58	11739.26	14.51	11212.70	14.42	11486.59	0.07	215.03
4	20.00	11341.52	19.57	2053.90	19.95	10517.31	0.19	4197.47	14.46	12183.52	14.50	10632.66	14.43	11551.48	0.03	636.73
5	20.09	11511.59	20.01	9088.68	19.99	8247.69	0.04	1383.67	14.58	12028.05	14.60	11013.83	14.43	11453.31	0.08	415.28
6	20.10	9478.05	20.01	10047.09	20.13	7176.36	0.05	1241.08	14.61	11591.16	14.20	10999.98	14.58	11099.79	0.19	258.39
7	20.29	8338.05	20.15	8902.26	20.18	8230.06	0.06	294.74	14.44	11512.60	14.05	11460.83	14.38	11368.05	0.17	59.80
8	20.39	8381.06	20.27	9938.98	20.29	10036.58	0.05	758.46	14.88	11948.74	14.75	11144.51	14.72	11113.16	0.07	386.72
9	20.35	11413.82	20.12	9267.01	20.12	9768.72	0.11	916.93	14.77	12617.22	14.62	11654.17	14.53	11830.74	0.10	418.62
10	19.55	10944.63	19.45	10556.67	19.63	10571.41	0.08	179.51	14.37	12094.18	13.96	11523.96	14.31	11727.84	0.18	235.92
11	19.88	6189.25	19.69	6290.30	20.07	10311.70	0.15	1919.97	14.61	12064.19	14.55	11189.35	14.59	11958.27	0.02	389.84
12	19.76	10085.27	20.21	10421.58	19.95	9926.64	0.19	206.35	14.37	12566.99	14.80	11919.14	14.34	12037.22	0.21	281.72
13	20.10	10429.08	19.91	10379.02	19.98	10291.90	0.08	56.68	14.38	11999.88	14.32	11566.11	14.34	11649.30	0.02	187.97
14	20.40	9039.01	20.27	8225.49	20.34	9885.17	0.05	677.60	13.96	12284.03	14.02	11114.65	13.95	11847.98	0.03	482.51
15	20.76	8126.31	21.03	9891.36	20.96	8498.57	0.12	759.66	14.42	11648.58	14.65	12458.08	14.43	11700.07	0.11	370.06
16	20.11	10006.95	20.13	7924.33	19.62	6329.16	0.24	1505.84	14.38	11752.57	14.59	11690.26	13.88	11222.92	0.30	236.37
17	21.25	8080.89	21.19	7404.86	21.34	7611.61	0.06	282.84	13.93	12941.97	13.84	12460.54	13.86	12528.32	0.04	212.78
18	20.23	8060.08	20.40	8893.51	20.41	7343.26	0.08	633.49	13.90	12355.32	14.31	12072.26	13.93	12012.74	0.19	149.45
19	19.99	9801.98	19.75	3899.38	20.15	9505.52	0.17	2715.34	14.25	12426.51	13.93	11790.36	13.97	12253.72	0.14	268.59
20	20.14	6492.59	21.62	9994.27	20.26	8118.27	0.67	1430.77	14.32	12257.20	15.64	12169.35	14.34	12443.07	0.62	114.11
21	20.17	5953.07	20.55	5424.51	20.66	8589.56	0.21	1384.36	13.45	11966.00	13.95	11884.13	13.96	12276.40	0.24	168.96
22	20.13	8729.56	20.13	8303.81	19.97	10218.65	0.08	820.92	13.98	12093.07	13.95	11824.93	13.72	12668.96	0.12	352.12
23	20.22	9378.74	20.00	10149.44	20.36	8465.27	0.15	688.38	14.24	12904.39	13.82	11976.77	14.11	12134.16	0.17	405.31
24	20.38	7646.68	20.28	10138.39	20.39	9424.71	0.05	1047.71	14.31	11925.96	14.04	12130.84	14.06	11990.45	0.13	85.54
25	19.66	5621.64	20.22	8753.24	20.04	9897.92	0.24	1807.51	14.45	12184.17	14.81	11835.12	14.53	11917.43	0.15	148.98
26	21.29	617.83	21.13	7546.53	21.07	7871.13	0.10	3345.36	14.51	11833.62	14.67	11925.48	14.49	11783.97	0.08	58.62
27	19.45	6621.96	19.82	8860.00	19.90	10455.05	0.20	1572.17	13.84	11964.24	14.02	11636.72	13.97	12414.52	0.08	318.85
28	20.12	9371.15	20.13	9140.53	20.17	10346.02	0.02	522.47	14.24	12965.41	14.00	12446.34	14.26	12501.93	0.12	232.70

	H. armigera probe								Control probe							
	Technical Rep. 1		Technical Rep. 2		Technical Rep. 3				Technical Rep. 1		Technical Rep. 2		Technical Rep. 3			
Sample	Cq	End RFU	Cq	End RFU	Cq	End RFU	SD Cq	SD End RFU	Cq	End RFU	Cq	End RFU	Cq	End RFU	SD Cq	SD End RFU
29	19.55	10542.64	19.32	9098.62	19.15	4195.87	0.16	2716.28	13.61	12803.84	13.74	12081.56	13.52	11777.92	0.09	430.30
30	20.45	9801.09	20.23	8410.55	20.22	6906.82	0.10	1181.88	14.35	12679.56	14.00	12307.58	13.96	11643.35	0.18	428.60
31	20.32	7591.59	20.28	8588.59	20.42	8984.37	0.06	585.99	13.93	12343.47	14.03	11968.44	14.03	12518.98	0.05	229.63
32	20.07	7477.87	20.02	10674.61	20.09	7768.63	0.03	1443.32	14.41	12450.26	14.04	12168.18	14.42	12207.75	0.18	124.70
33	20.46	7695.02	20.49	8820.71	20.33	5730.50	0.07	1276.98	14.26	12578.13	14.01	12304.78	13.87	12135.99	0.16	182.18
34	20.99	5210.57	20.92	7571.08	20.95	7101.77	0.03	1020.29	13.83	12314.06	13.84	12015.18	13.77	12100.58	0.03	125.70
35	20.95	9037.31	20.39	9194.65	20.49	8652.75	0.24	227.62	13.88	13185.77	13.60	12778.04	13.66	12957.54	0.12	166.85
36	22.11	6178.55	22.06	7849.53	22.19	8124.06	0.05	859.75	14.56	13074.77	14.48	12671.96	14.63	13095.44	0.06	194.94
37	20.05	8192.79	19.95	6090.00	20.27	8735.98	0.13	1141.05	13.64	13429.69	13.70	12324.13	13.64	12466.26	0.03	491.10
38	20.76	7469.11	20.97	8113.62	21.12	8465.02	0.15	412.41	13.70	12867.41	13.77	12633.26	13.88	12721.17	0.07	96.57
39	19.53	9024.36	19.97	11255.70	19.79	10513.89	0.18	927.84	13.50	12651.66	13.84	12991.91	13.58	12896.79	0.14	143.34
40	21.23	9402.32	21.27	8699.24	21.26	8813.20	0.02	308.11	14.27	12996.35	14.06	12698.18	13.96	12479.87	0.13	211.69
41	19.96	10011.06	20.01	10663.25	19.29	8582.10	0.33	869.13	13.86	12900.68	13.77	12455.17	13.70	12589.11	0.07	186.64
42	20.44	6803.98	20.30	8117.57	20.65	8617.07	0.14	764.66	14.05	12485.37	13.86	12434.33	14.35	12284.33	0.20	85.33
43	20.43	8523.72	20.32	8972.16	20.41	9513.12	0.05	404.51	13.71	13190.78	13.68	12591.50	13.80	13433.14	0.05	353.75
44	19.36	9966.05	18.96	1218.38	19.33	9990.56	0.18	4129.48	13.77	13250.30	13.66	11466.77	13.78	12787.94	0.05	755.74
45	19.16	9879.53	19.64	9713.85	19.60	9332.08	0.22	229.23	13.56	13124.94	14.32	12567.97	14.04	12318.26	0.32	337.20
46	20.56	5917.50	20.84	2366.34	21.08	5589.27	0.21	1602.28	13.65	12641.44	14.03	11748.78	14.07	12252.98	0.19	365.44
47	19.43	7608.64	19.73	10385.04	19.65	9919.40	0.13	1214.03	13.74	12367.35	13.91	12639.83	13.75	13120.11	0.08	311.19
48	20.01	9051.96	20.13	8735.87	20.16	8130.17	0.06	382.46	13.96	12733.68	14.08	12639.28	14.41	12386.41	0.19	146.61
49	19.12	9337.92	19.11	11082.28	19.31	11035.37	0.09	811.47	13.86	12731.83	13.80	12528.25	13.86	13225.28	0.03	292.65
50	20.04	10065.43	19.87	9340.20	19.79	9223.55	0.11	372.43	14.29	13061.68	13.75	12492.05	14.02	12512.51	0.22	263.84
51	20.20	8470.29	20.09	9180.35	20.25	8498.40	0.06	328.30	13.86	13228.03	13.65	12380.45	13.80	13016.47	0.09	360.19
52	20.02	9865.93	20.09	10119.04	20.11	10444.21	0.04	236.69	14.29	12764.93	14.32	12465.49	14.31	13287.12	0.01	339.51
53	20.07	7777.45	20.28	9493.99	20.13	9889.50	0.08	916.74	13.65	12743.80	13.80	12740.50	13.66	12983.44	0.07	113.75
54	19.52	10217.80	20.07	7626.25	20.08	9288.87	0.26	1072.04	13.47	13065.10	13.86	12558.32	13.85	13283.54	0.18	303.77
55	20.06	10428.07	19.72	2345.42	19.83	9993.53	0.14	3712.02	14.04	12793.54	13.97	11790.00	13.82	12723.13	0.09	457.38
56	20.17	10206.95	19.97	8583.01	19.95	4970.54	0.10	2188.53	13.90	12988.43	13.74	12845.54	13.85	11977.15	0.07	446.87
57	21.45	5551.14	21.51	7383.00	21.57	8225.53	0.05	1116.44	14.46	12861.03	14.44	12388.64	14.45	12939.61	0.01	243.33
58	19.72	9903.67	19.75	10274.81	19.90	10228.41	0.08	165.11	14.29	12666.31	14.28	12376.17	14.31	12461.33	0.01	121.77
59	19.15	11490.63	19.12	11410.40	18.75	11187.21	0.18	128.37	14.02	13155.18	14.02	12365.98	13.56	12711.26	0.22	323.03

	H. armigera probe								Control probe							
	Technical Rep. 1		Technical Rep. 2		Technical Rep. 3				Technical Rep. 1		Technical Rep. 2		Technical Rep. 3			
Sample	Cq	End RFU	Cq	End RFU	Cq	End RFU	SD Cq	SD End RFU	Cq	End RFU	Cq	End RFU	Cq	End RFU	SD Cq	SD End RFU
60	19.03	10035.60	19.21	11136.88	19.31	10979.53	0.11	486.32	13.74	12678.20	13.93	12727.13	13.82	12842.08	0.07	68.69
61	20.21	9695.37	20.18	9461.39	20.26	9994.44	0.04	218.16	13.74	13227.38	13.82	12518.89	13.70	12917.07	0.05	289.98
62	20.70	9592.10	20.94	9475.16	20.71	9552.68	0.11	48.58	14.47	12991.00	14.51	12299.05	14.41	12931.50	0.04	313.10
63	20.05	9424.43	19.60	8468.24	20.05	10248.98	0.21	727.64	13.94	12620.84	13.82	12153.43	13.87	12644.36	0.05	226.09
64	20.29	9942.74	19.77	8362.74	20.15	5712.11	0.22	1745.49	14.05	13083.40	13.50	12348.96	14.03	12002.11	0.26	450.79
65	19.85	10827.85	19.23	9117.08	19.39	5021.22	0.26	2436.29	14.43	13179.29	13.77	11594.87	13.87	12270.54	0.29	649.17
66	20.13	9289.06	20.03	8388.36	20.27	9751.04	0.10	565.84	13.87	12864.97	13.71	11931.77	13.84	12707.92	0.07	407.97
67	19.27	10862.39	19.40	10141.42	19.21	6427.50	0.08	1943.12	14.35	12783.55	14.39	12496.47	14.31	12111.62	0.03	275.28
68	20.34	6966.95	20.43	8607.41	20.31	6664.40	0.05	853.61	14.29	13033.40	14.27	12359.73	13.94	12320.65	0.16	327.17
69	20.20	7208.27	20.03	7172.77	20.24	8366.85	0.09	554.72	13.90	12626.35	13.85	11885.09	14.19	12836.10	0.15	407.96
70	19.49	7187.15	19.38	8811.15	19.34	1239.92	0.06	3254.57	13.67	13050.94	13.60	12021.35	13.64	11676.45	0.03	583.88

Table S2. Analysis of Variance table comparing control probe Cq values across treatments.

	Df	Sum Sq	Mean Sq	F value	Pr (>F)
**Treatment**	2.00	14.14	2.07	643.60	9.99e-08 ***
**Residuals**	6.00	0.02	0.00		

*** indicates p<0.0001

Table S3. Tukey HSD Test Results table comparing control probe Cq values across treatments.

				**95% CI**	
**Treatment**	**Mean Difference**	**Std. Error**	**p-value**	**Lower Bound**	**Upper Bound**
Pos Ctrl-Pos Ctrl BP	1.065081	0.0327261	1.20E-06	0.9230763	1.2070858
RC buffer-Pos Ctrl	-0.5706772	0.0327261	4.28E-05	-0.7126819	-4.29E-01
RC buffer-Pos Ctrl BP	-1.6357582	0.0327261	1.00E-07	-1.7777629	-1.4937535

Table S4. Analysis of Variance table comparing *H. armigera* probe RFU values across treatments

	Df	Sum Sq	Mean Sq	F value	Pr (>F)
**Treatment**	2	15,923,409	7,961,705	15.71	0.00413 **
**Residuals**	6	3,041,807	506,968		

*** indicates p<0.0001

Table S5. Tukey HSD Test Results table comparing *H. armigera* probe RFU values across treatments.

Treatment	Mean Difference	Std. Error	p-value	95% CI Lower Bound	95% CI Upper Bound
Pos Ctrl-Pos Ctrl BP	3228.999	411.083	0.0034754	1445.2299	5012.769
RC buffer-Pos Ctrl	1991.165	411.083	0.0324605	207.3955	3774.9346
RC buffer-Pos Ctrl BP	-1237.834	411.083	0.1638087	-3021.6039	545.9352

Table S6. Analysis of Variance table comparing control probe RFU values across treatments.

	Df	Sum Sq	Mean Sq	F value	Pr (>F)
**Treatment**	2.00	14335.00	7167.00	0.10	0.91
**Residuals**	6.00	429429.00	71572.00		

*** indicates p<0.0001
